# Tumor-infiltrating FoxP3^+^ Tregs predict favorable outcome in colorectal cancer patients: A meta-analysis

**DOI:** 10.18632/oncotarget.17722

**Published:** 2017-06-07

**Authors:** Guoming Hu, Zhi’an Li, Shimin Wang

**Affiliations:** ^1^ Department of General Surgery (Breast and Thyroid Surgery), Shaoxing People’s Hospital, Shaoxing Hospital of Zhejiang University, 312000, Shaoxing, China; ^2^ Department of Surgical Oncology, Shaoxing Second Hospital, 312000, Shaoxing, China; ^3^ Department of Nephrology, Shaoxing People’s Hospital, Shaoxing Hospital of Zhejiang University, 312000, Shaoxing, China

**Keywords:** tumor-infiltrating FoxP3^+^ Tregs, favorable outcome, human colorectal cancer, meta-analysis

## Abstract

FoxP3^+^ regulatory T cells (FoxP3^+^ Tregs) are considered to be a key mediator in immune escape and tumor progression. However, the role of FoxP3^+^ Tregs in human colorectal cancer (CRC) remains controversial. Herein, we conducted a meta-analysis including 17 published studies with 3811 patients identified from PubMed and EBSCO to assess the prognostic impact of tumor-infiltrating FoxP3^+^ Tregs in human CRC. We found FoxP3^+^ Tregs infiltrating into both intraepithelium and stroma within tumor were significantly positively correlated with 1, 3, 5 and 10-year overall survival (OS), but not with 1, 3, 5-year disease-free survival (DFS) of patients. Interestingly, in stratified analyses by compartments within tumor FoxP3^+^ Tregs infiltrating into, FoxP3^+^ Tregs invading stromal compartment significantly improved 3 and 5-year OS, yet OS wasn’t improved when FoxP3^+^ Tregs infiltrated into intraepithelium only. Furthermore, FoxP3^+^ Tregs invading both intraepithelium and stroma significantly inversely correlated with TNM stage of CRC. In conclusion, High density of FoxP3^+^ Tregs within tumor especially at stromal compartment leads to a favorable outcome in CRC, implicating FoxP3^+^ Tregs are one of valuable indexes for prognostic prediction in human CRC.

## INTRODUCTION

Human colorectal cancer (CRC) is one of the most common fatal malignancies worldwide. A large amount of evidence has demonstrated that tumor microenvironment (TME) linked closely with the initiation, promotion, and progression of CRC via diverse mechanisms such as stimulating angiogenesis and immune suppression [1]. Immune cells are the core components of the TME [2]. Regulatory T cells (Tregs) are considered to be a key mediator in subverting antitumor immune responses and subsequently promote tumor progression.

The transcription factor forkhead box P3 (FoxP3), an intracellular key molecule for Tregs development and function [3], is considered to represent the most specific Treg cell marker so far [4]. Recent studies have demonstrated that increased proportion of tumor-infiltrating FoxP3^+^ Tregs predict a poor prognosis of patients with cancer, including breast [5], ovarian [6], hepatocellular [7] and gastric carcinomas [8]. However, in human CRC, high density of tumor-infiltrating FoxP3^+^ Tregs was shown to associate with worse survival in several studies, [9, 10] while some other studies reported opposite results [11, 12]. Thus, a deep understanding is warranted. Moreover, the potential of FoxP3^+^ Tregs as an effective index in prognostic prediction and targeted immunotherapy is necessary to be explored.

Here, we performed this meta-analysis to test overall survival (OS) and disease-free survival (DFS) as outcomes in human CRC with known FoxP3^+^ Tregs density according to the compartment within tumor they invade. The aim of this study was to quantitatively summarize the association between high FoxP3^+^ Tregs infiltration and clinical outcomes in human CRC, and thereby provided more evidence on the clinical value of FoxP3^+^ Tregs as a prognostic index and immunotherapeutic target for human CRC.

## RESULTS

### Search results and description of studies

**Figure 1:**
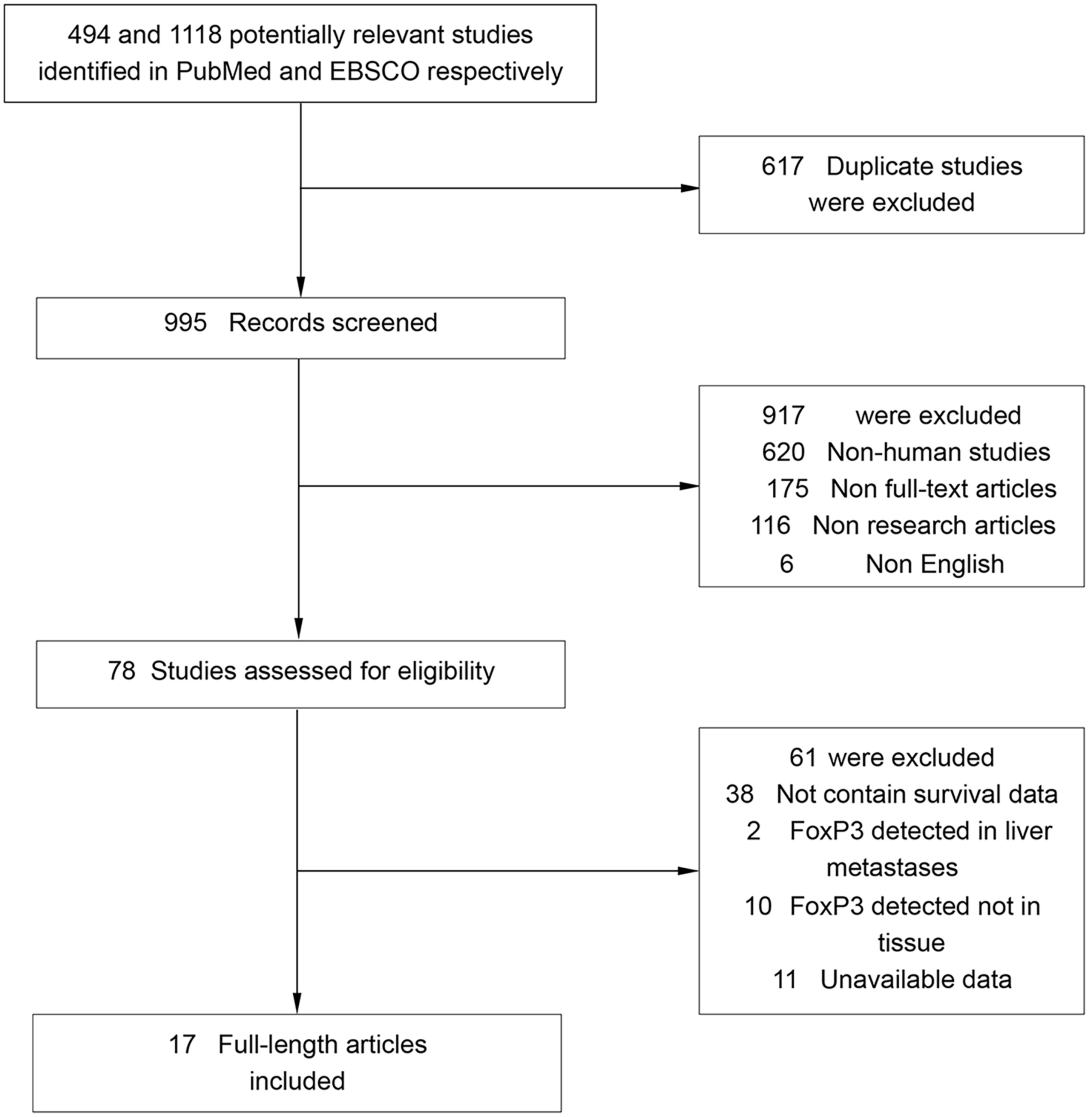
Flow chart diagram of study selection

Literature searches yield total 1612 records and the results are shown in Figure 1. 17 studies including 3811 patients were identified for the assessment of intratumoral FoxP3^+^ Tregs density [12–28]. All the studies were evaluated by the Newcastle–Ottawa Scale (NOS), and were in accordance with the inclusion criteria and suitable for data consolidation. Characteristics of included studies for OS, DFS (or RFS) and clinicopathological features such as TNM stage were shown in Table 1 and Supplementary Table 1 respectively.

**Table 1:** Main characteristics of the included studies

Location of FoxP3^+^ Tregs	Study	Year	No. of Patients	Cut-off for high density	FoxP3^+^ Tregs: high / low	Tumor stage	Follow-up years: median (range)	Survival	Quality Score (NOS)
Both intraepithelial and stromal compartments	Suzuki, H. etal [13]	2010	94	mean	30/64	I-IV	NR	OS, DFS	7
	Reimers, M. S. etal [14]	2014	478	median	238/240	I-IV	NR	OS, DFS	6
	Ling, A. etal [12]	2014	204	other	125/79	I-IV	9.42	OS	8
	Chen, J.X. etal [15]	2014	102	other	47/55	I-IV	NR	OS	7
	Frey, D. M. etal [16]	2010	1232	median	614/618	NR	NR	OS	6
	Suzuki, H. etal [17]	2013	88	mean	34/54	I-IV	NR	OS, DFS	7
	Kim, M. etal [18]	2013	65	mean	27/38	I-IV	≥ 5	OS	7
	Hanke, T. etal [19]	2015	820	mean	34/786	II	3.83 (0.08, 12.67)	OS	6
	Vlad, C. etal [21]	2015	42	median	21/21	II, III	NR	OS	6
	Xu, W. etal [20]	2013	90	median	21/69	I-IV	5.42 (5.25, 6.08)	OS	7
	Tosolini, M. etal [28]	2011	56	mean	18/38	NR	NR	DFS	6
Intraepithelial compartment	Sinicrope, F. A. etal [22]	2009	160	other	101/59	II, III	NR	OS, DFS	7
	Lee, W. S. etal [23]	2010	63	mean	39/24	II	10.42 (3.54, 14.04)	OS, DFS	8
	Nosho, K. etal [25]	2010	768	median	384/384	I-IV	11.6	OS	7
	Zeestraten, E. C. etal [24]	2013	76	median	36/40	I-III	7.3 (0.1, 23.1)	OS, DFS	7
	Shinto, E. etal [26]	2014	81	median	36/45	II, III	4.60 (1.68, 7.62)	OS	8
	Xu, W. etal [20]	2013	90	median	13/77	I-IV	5.42 (5.25, 6.08)	OS	7
Stromal compartment	Sinicrope, F. A. etal [22]	2009	160	other	118/42	II, III	NR	OS, DFS	7
	Ling, A. etal [12]	2014	405	other	151/254	I-IV	9.42	OS	8
	Lee, W. S. etal [23]	2010	63	mean	39/24	II	10.42 (3.54, 14.04)	OS, DFS	8
	Yoon, H. H. etal [27]	2012	156	other	78/78	II, III	8 (4.7, 8.0)	OS	6
	Xu, W. etal [20]	2013	90	median	21/69	I-IV	5.42 (5.25, 6.08)	OS	7
	Shinto, E. etal [26]	2014	81	median	36/45	II, III	4.60 (1.68, 7.62)	OS	8

### Meta-analyses

#### Density of FoxP3^+^ Tregs both in stromal and intraepithelial compartments in tumor tissue

#### OS

**Figure 2:**
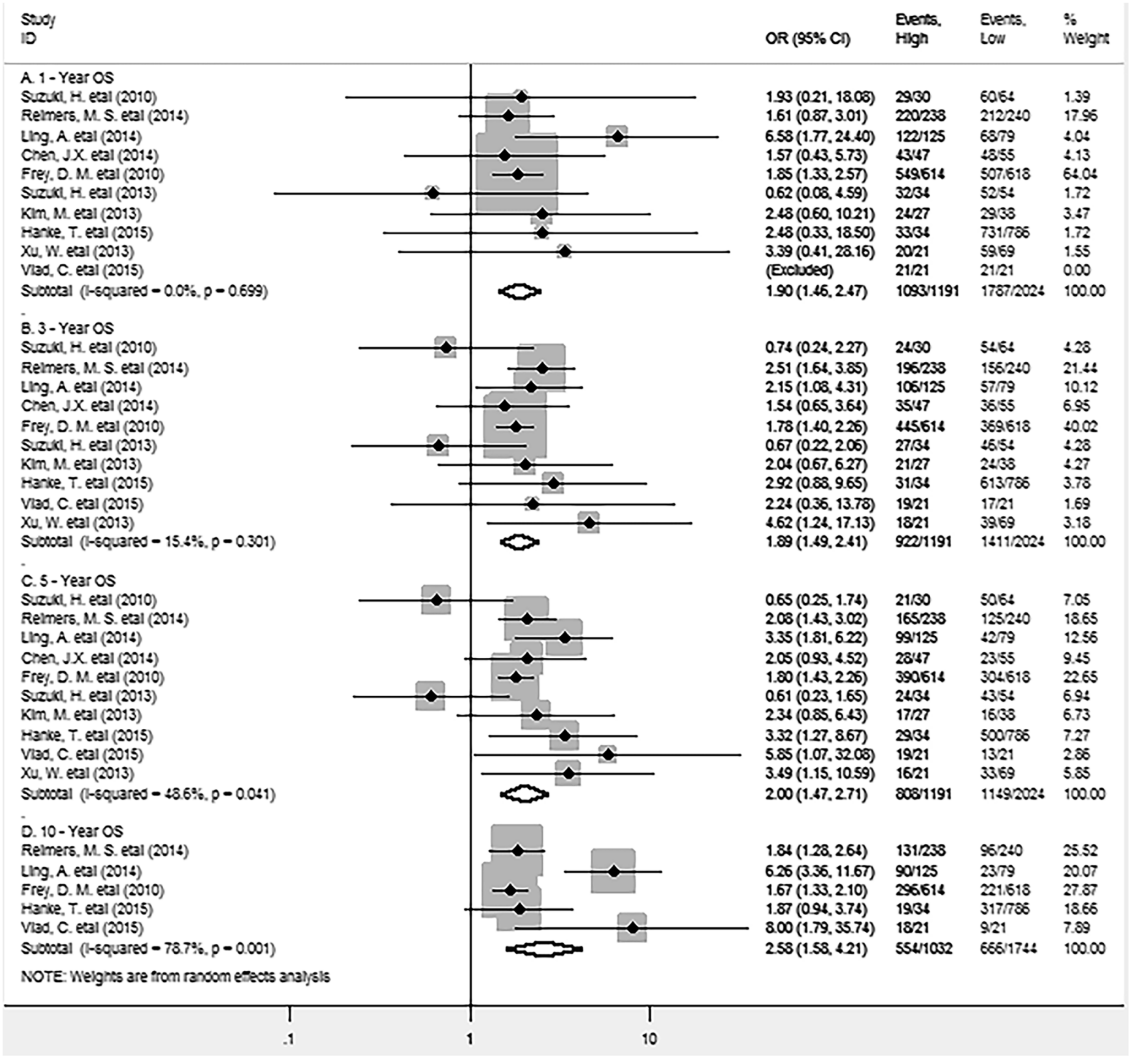
Forest plots describing OR of the association between FoxP3^+^ Tregs infiltrating into both stroma and intraepithelium and OS at 1, 3, 5 and 10-year

In this meta-analysis, as shown in Figure 2, pooled results indicated that high density of FoxP3^+^ Tregs was significantly associated with improved 1-year (OR = 1.90, 95% CI = 1.46 to 2.47, P = 0.000), 3-year (OR = 1.89, 95% CI = 1.49 to 2.41, P = 0.000) and 5-year OS (OR = 2.00, 95% CI = 1.47 to 2.71, P = 0.000) of patients. There was no significant heterogeneity among studies. Similar result was observed in analysis between FoxP3^+^ Tregs and 10-year OS rate (OR = 2.58, 95% CI = 1.58 to 4.21, P = 0.000), with significant heterogeneity being observed among studies.

#### DFS

**Figure 3:**
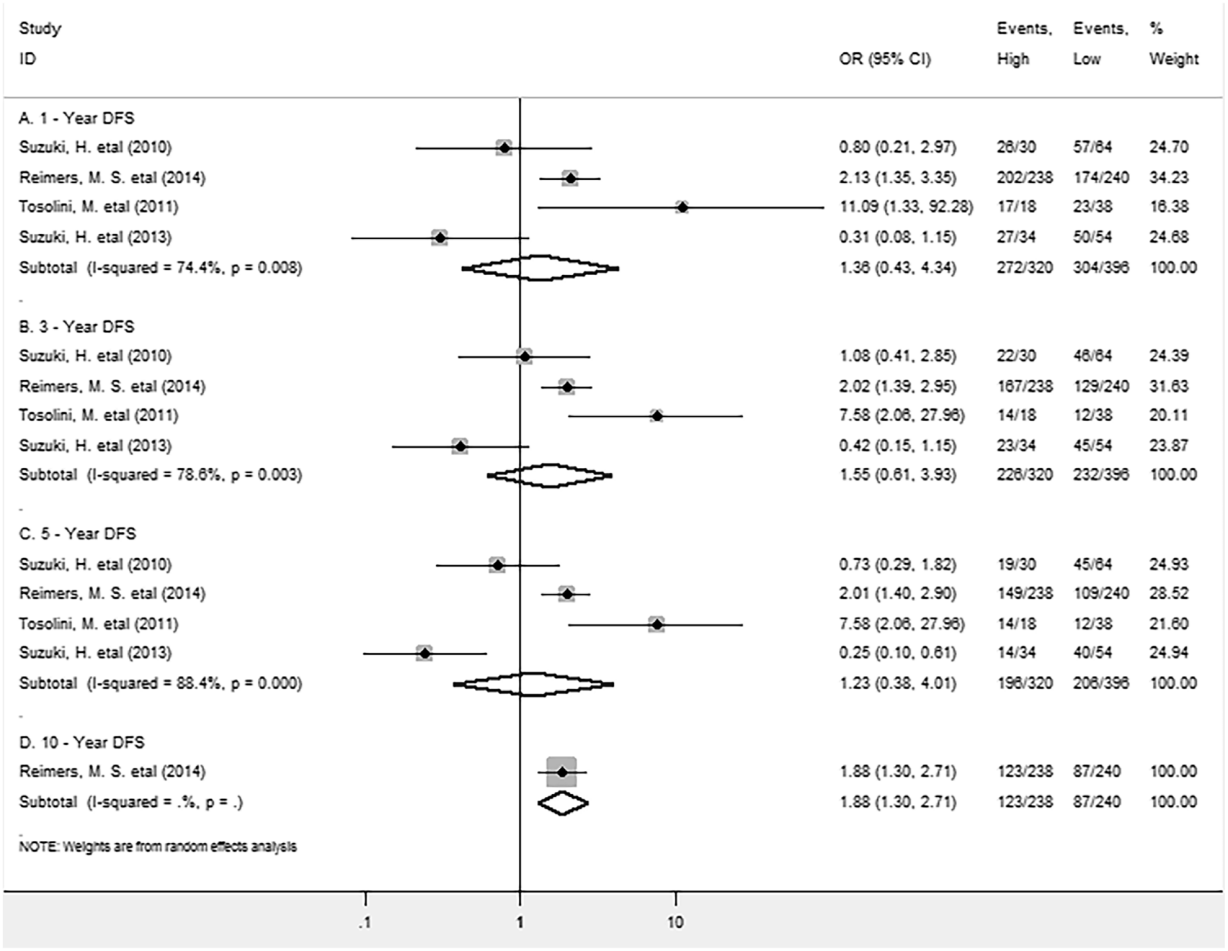
Forest plots describing OR of the association between FoxP3^+^ Tregs infiltrating into both stroma and intraepithelium and DFS at 1, 3, 5 and 10-year

The meta-analysis of all these studies showed that there were no significant association between FoxP3^+^ Tregs and 1, 3, 5-year DFS (OR = 1.36, 95% CI = 0.43 to 4.34, P = 0.604; OR = 1.55, 95% CI = 0.61 to 3.93, P = 0.353 and OR = 1.23, 95% CI = 0.38 to 4.01, P = 0.731 respectively) of CRC patients. Significant heterogeneity among studies was observed in each analysis (Figure 3).

#### Intraepithelial FoxP3^+^ Tregs density in tumor tissue

#### OS

**Figure 4:**
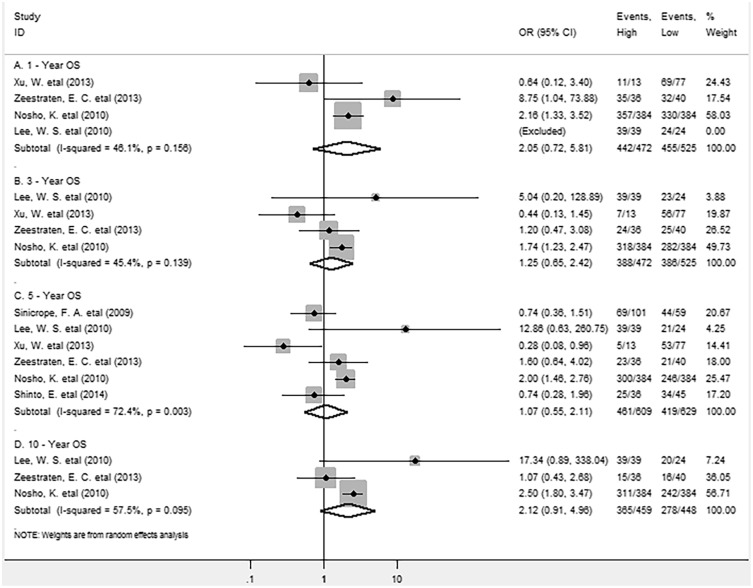
Forest plots describing OR of the association between intraepithelial FoxP3^+^ Tregs and OS at 1, 3, 5 and 10-year

As for FoxP3^+^ Tregs density in intraepithelial compartment in tumor tissue, pooled results showed no significant association between FoxP3^+^ Tregs and survival, high density of FoxP3^+^ Tregs in intraepithelium wasn’t significantly associated with longer 1-year (OR =2.05, 95% CI = 0.72 to 5.81, P = 0.176) or 3-year OS (OR = 1.25, 95% CI = 0.65 to 2.42, P = 0.508) (Figure 4). Similar results were observed in analyses between FoxP3^+^ Tregs infiltration and 5-year (OR = 1.07, 95% CI = 0.55 to 2.11, P = 0.835) or 10-year OS rate (OR = 2.12, 95% CI = 0.91 to 4.96, P = 0.084).

#### DFS

Similarily, no significant association was found between intraepithelial FoxP3^+^ Tregs and 1-year (OR = 1.52, 95% CI = 0.74 to 3.16, P = 0.257), 3-year (OR = 0.98, 95% CI = 0.57 to 1.67, P = 0.930), 5-year DFS (OR = 1.24, 95% CI = 0.76 to 2.05, P = 0.388) or 10-year DFS (OR = 1.80, 95% CI = 0.84 to 3.85, P = 0.128). (Supplementary Figure 1)

#### Stromal FoxP3^+^ Tregs density in tumor tissue

#### OS

**Figure 5:**
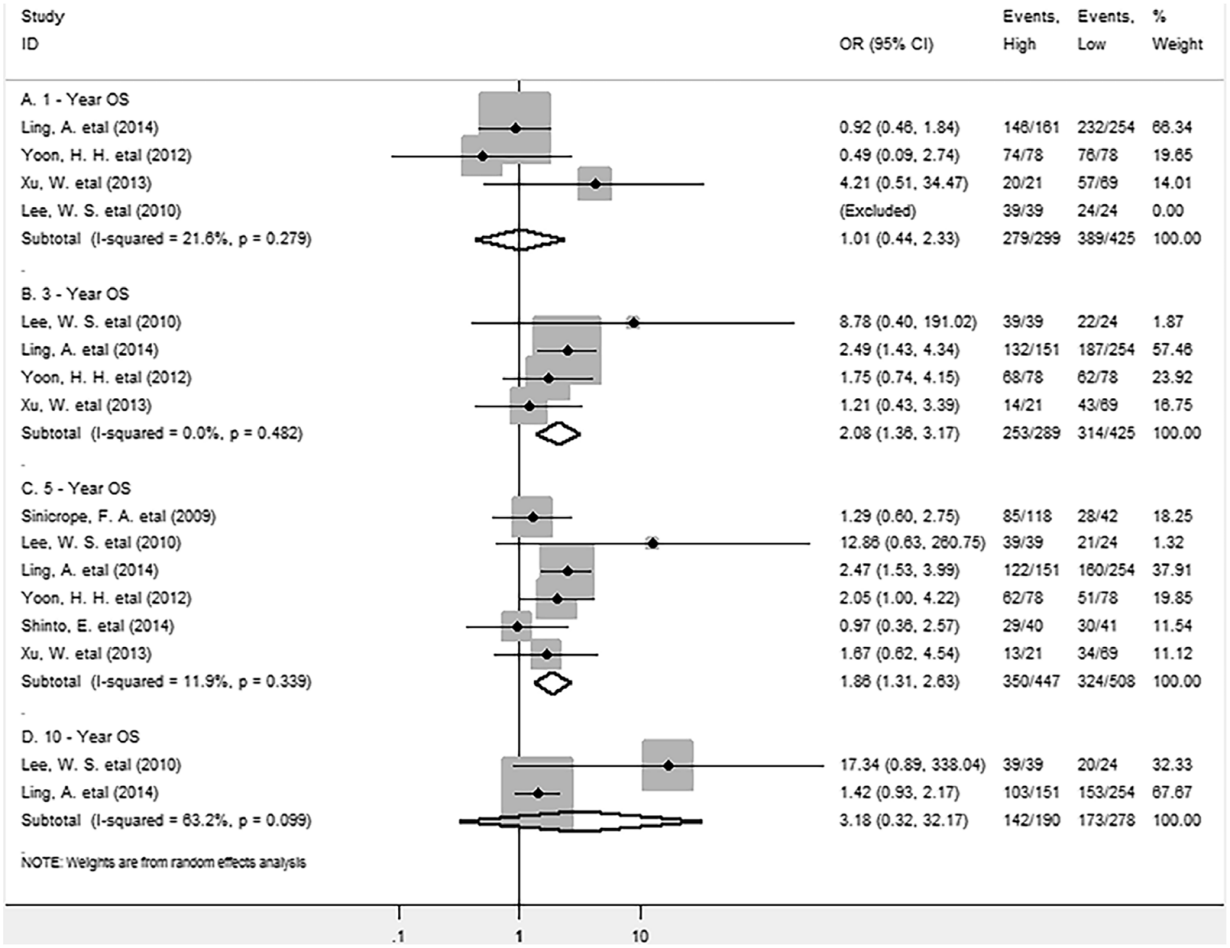
Forest plots describing OR of the association between stromal FoxP3^+^ Tregs and OS at 1, 3, 5 and 10-year

As for FoxP3^+^ Tregs in the stromal compartment, the meta-analysis showed that there was no significant association between FoxP3^+^ Tregs and 1-year (OR = 1.01, 95% CI = 0.44 to 2.33, P = 0.987) or 10-year (OR = 3.18, 95% CI = 0.32 to 32.17, P = 0.326) OS; whereas FoxP3^+^ Tregs infiltration significantly improved 3-year (OR = 2.08, 95% CI = 1.36 to 3.17, P = 0.001) and 5-year (OR = 1.86, 95% CI = 1.31 to 2.63, P = 0.000) OS for CRC patients, with no significant heterogeneity existing among studies (Figure 5).

#### DFS

As for the relationship between FoxP3^+^ Tregs and DFS, the meta-analysis showed high density of stromal FoxP3^+^ Tregs was not associated with improved 5-year DFS (OR = 1.75, 95% CI = 0.50 to 6.08, P = 0.121) of patients (Supplementary Figure 2).

In addition, we found that FoxP3^+^ Tregs infiltrating into both stromal and intraepithelial compartments was significantly associated with early TNM stage (I+II) of CRC (OR = 2.95, 95% CI = 1.81 to 4.83, P = 0.000), whereas no significant association was observed between intraepithelial or stromal FoxP3^+^ Tregs and TNM stage (OR = 0.99, 95% CI = 0.33 to 3.01, P = 0.908; OR = 1.57, 95% CI = 0.84 to 2.92, P = 0.158) (Supplementary Figure 3).

### Sensitivity analysis

Sensitivity analyses were used to determine the influence of individual studies on the overall OR. As a result, the plot showed that all the individual studies had no important impact on the results for 1, 3, 5 or 10-year OS, which correlating with FoxP3^+^ Tregs both in stroma and intraepithelium within tumor (Supplementary Figure 4).

### Publication bias

**Figure 6:**
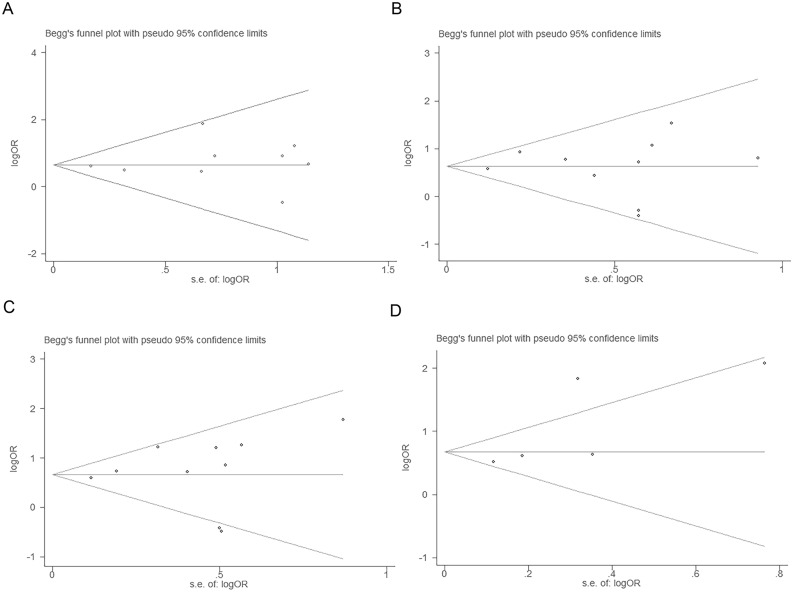
Tests for publication bias for OR of OS at 1, 3, 5 and 10-year (A, B, C and D respectively)

Funnel plot and Egger’s test were performed to assess the publication bias of this meta-analysis. No publication bias existed between FoxP3^+^ Tregs infiltrating into both stroma and intraepithelium and 1, 3, 5 or 10-year OS (continuity corrected *P* = 0.569; *P* = 0.913; *P* = 0.717 and *P* = 0.152 >0.05 respectively) (Figure 6) or DFS (data not shown).

## DISCUSSION

FoxP3^+^ Tregs are highly enriched in the TME and are considered to be a pivotal mediator of immune suppression, therefore facilitating tumor progression [31-32]. However, FoxP3^+^ Tregs shows its anti-tumor effect in several cancers. Previous meta-analyses reported that increased tumor-infiltrating FoxP3^+^ Tregs improved OS in human CRC [29, 30]. However, the studies included in these meta-analyses only reported the data on FoxP3^+^ Tregs either from stroma, intraepithelium or both sites. Thus, the results were not accurate even wrong when they were from the combinations of all these studies.

In this meta-analysis, we found FoxP3^+^ Tregs invading both stroma and intraepithelium within tumor were significantly positively correlated with 1, 3, 5 and 10-year OS, but not with 1, 3, 5 or 10-year DFS in CRC. In addition, FoxP3^+^ Tregs infiltration was significantly inversely associated with TNM stage of CRC. Interestingly, FoxP3^+^ Tregs infiltrating into different sites (intraepithelium or stroma) seemed to predict differential clinical outcomes as stated above, implicating FoxP3^+^ Tregs may have distinct roles depending upon their localization. The possible explanation was that FoxP3^+^ Tregs in stroma mainly inhibited the inflammatory anti-microbial response which facilitating tumor progression; whereas FoxP3^+^ Tregs in intraepithelium may inhibit anti-tumor immunity and promote tumor immune evasion maybe through the direct contact with tumor cells. We believe that our study provides significative statistical evidence to unravel the differential prognostic value of FoxP3^+^ Tregs in different locations in human CRC for the first time.

Several limitations should be noted from this meta-analysis. First, we can’t get pooled result as there is only one study included in some analyses. Second, significant heterogeneity observed across studies in some analyses can’t be completely accounted despite the use of random-effect models. Third, morphometric analysis for FoxP3+ Tregs used in included studies are not inconsistent. Finally, studies with negative results or small sample size may not be published, which can cause publication bias.

In conclusion, high density of FoxP3^+^ Tregs within tumor especially at stromal compartment leads to a favorable clinical outcome of CRC, implicating FoxP3^+^ Tregs are one of potential indexes for prognostic prediction and agonists through promoting FoxP3^+^ Tregs generation may be promising in immunotherapy for human CRC.

## MATERIALS AND METHODS

### Search strategy

We searched PubMed and EBSCO for studies assessing the density of FoxP3^+^ Tregs in tumor tissue and survival in CRC patients from 1996 to October 2016. The searching keywords were (“regulatory T cells” OR “Tregs” OR “FoxP3”) AND (“colorectal”) AND (“neoplasms” OR “cancer” OR “tumor” OR “carcinoma”). A total of 494 and 1118 entries were identified in PubMed and EBSCO respectively.

### Inclusion and exclusion criteria

Inclusion criteria of the meta-analysis were: studies included must have (1) been published as original articles; (2) evaluated human subjects; (3) FoxP3^+^ Tregs in tumor specimens was evaluated with immunohistochemical (IHC) method; (4) provided Kaplan – Meier curves of high and low FoxP3^+^ Tregs density with overall survival (OS), and/or disease-free survival (DFS), or relapse-free survival (RFS); (5) published in English.

We excluded studies that were not published as full texts, including commentary, conference abstracts and letters to editors, studies that not report sufficient data to estimate survival rates; studies that evaluated FoxP3^+^ Tregs with Flow Cytometry (FCM) or real-time reverse transcription polymerase chain reaction (RT-PCR), detected FoxP3^+^ Tregs in metastases and not in tumor tissues.

### Endpoints

OS and DFS (or RFS) are the endpoints used in this meta-analysis. OS was recorded as the primary endpoint, and the second endpoint was DFS (or RFS). Cut-offs of FoxP3^+^ Tregs density defined by individual studies classified CRC patients into high- and low- groups.

### Data extraction

Two authors (GM.H. and ZA.L.) independently reviewed and extracted data using predefined data abstraction forms from each eligible studies. Extracted information included first author’s name, publication year, country, number of patients, median age, gender, Tumor, Lymph Node, Metastasis (TNM) stage, tumor differentiation, time of follow-up, technique used to quantify FoxP3^+^ Tregs, and cut-off value to determine high FoxP3^+^ Tregs density. OS, DFS (or RFS) and clinicopathological data were extracted from the text, tables, or Kaplan – Meier curves for both high and low FoxP3^+^ Tregs density groups.

### Quality assessment

The studies included in the meta-analysis were cohort studies. The quality of each included study was assessed using Newcastle–Ottawa Scale (NOS) by two independent authors [33]. The studies with 6 scores or more were regarded as high quality studies. A consensus NOS score for each item was achieved.

### Statistical analysis

Extracted data were combined into a meta-analysis using STATA 12.0 analysis software (Stata Corporation, College Station, TX, USA). Statistical heterogeneity was assessed using the chi-squared based Q-test or the *I*^*2*^ method [34]. Data were combined according to the random-effect model in the presence of high heterogeneity [35], otherwise, the fixed-effect model was performed [36]. Sensitivity analysis was employed to assess the influence of each study on the pooled result. Begg’s funnel plot and Egger’s test [37] were calculated to investigate potential publication bias. All *P* values were two-sided and less than 0.05 are considered statistically significant.

## SUPPLEMENTARY MATERIALS FIGURES AND TABLE



## Abbreviations

FoxP3: forkhead box P3
Tregs: regulatory T cells
CRC: colorectal cancer
OS: overall survival
DFS: disease-free survival
OR: odds ratios
Cl: confidence interval
TNM: Tumor, Lymph Node, Metastasis
IHC: immunohistochemistry
FCM: Flow Cytometry
RT-PCR: real-time reverse transcription polymerase chain reaction
NR: not reported
