# Supplementary material for: Tumor-infiltrating FoxP3+ Tregs predict favorable outcome in colorectal cancer patients: A meta-analysis
Source: Oncotarget. 2017 Jun 7;8(43):75361–71. doi: 10.18632/oncotarget.17722 (PMC5650426; doi:10.18632/oncotarget.17722)
Supplement: Supplementary file 1 [file oncotarget-08-75361-s001.pdf]

Tumor-infiltrating FoxP3$^+$ Tregs predict favorable outcome in colorectal cancer patients: A meta-analysis

SUPPLEMENTARY MATERIALS

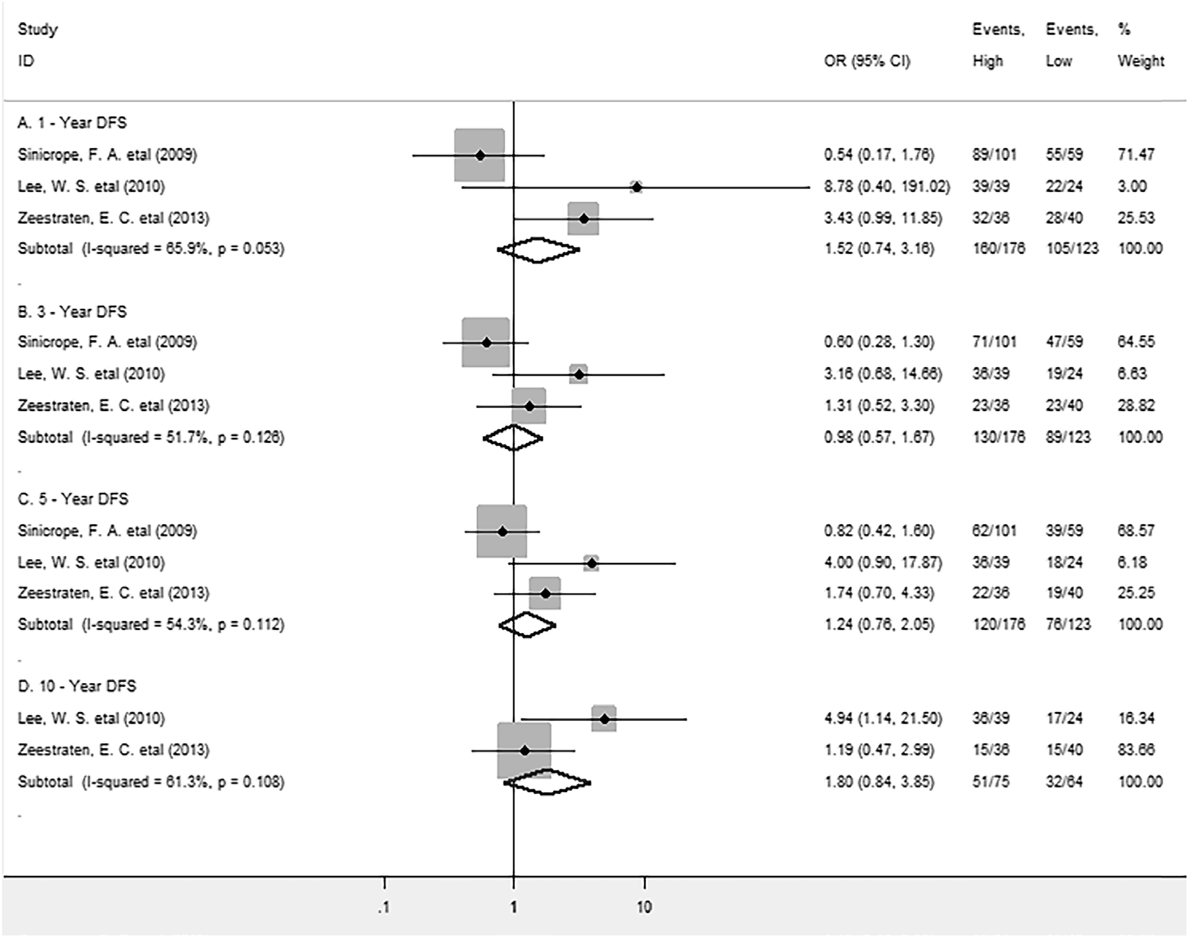

Supplementary Figure 1: Forest plots describing ORs of the association between intraepithelial FoxP3$^+$ Tregs and DFS at 1, 3, 5 and 10-year.

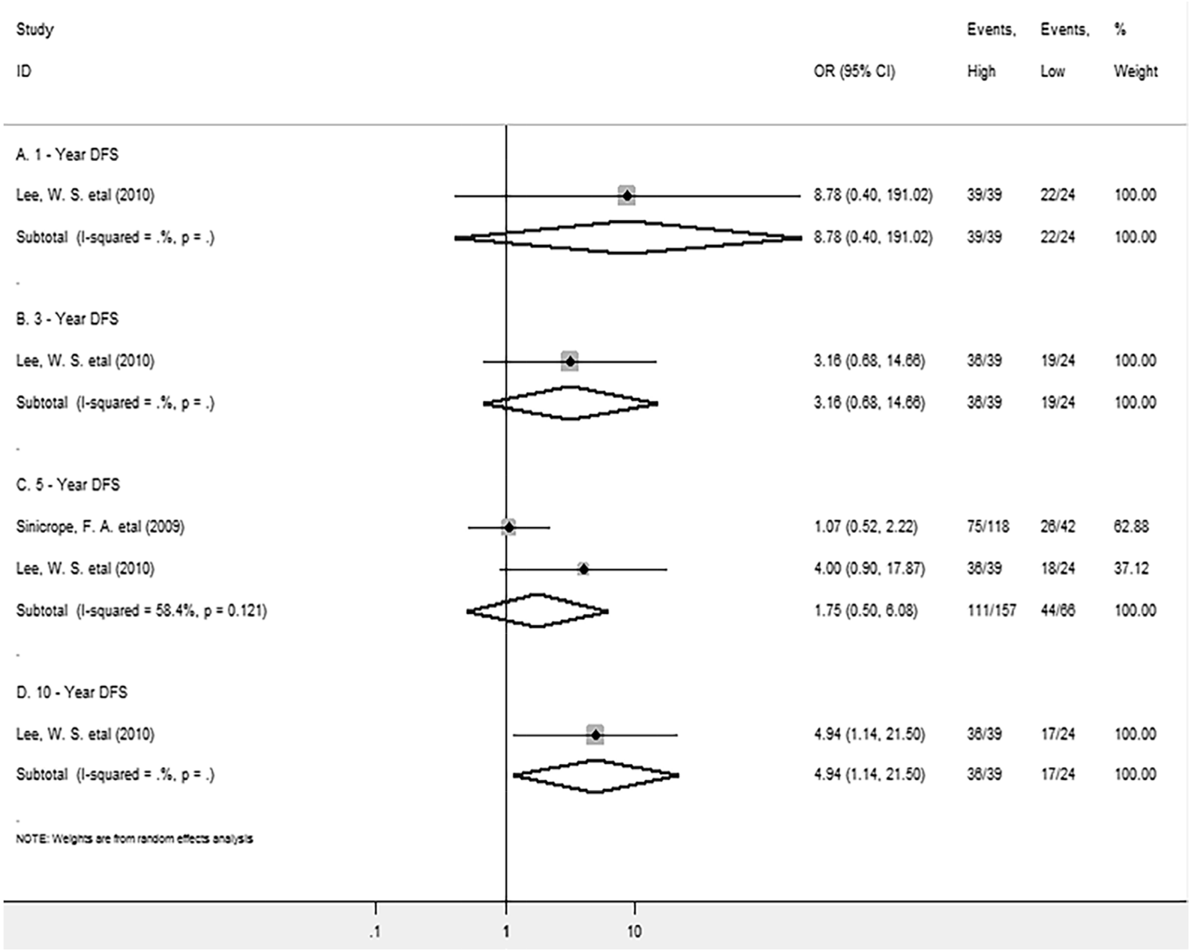

Supplementary Figure 2: Forest plots describing ORs of the association between stromal FoxP3$^+$ Tregs and DFS at 1, 3, 5 and 10-year.

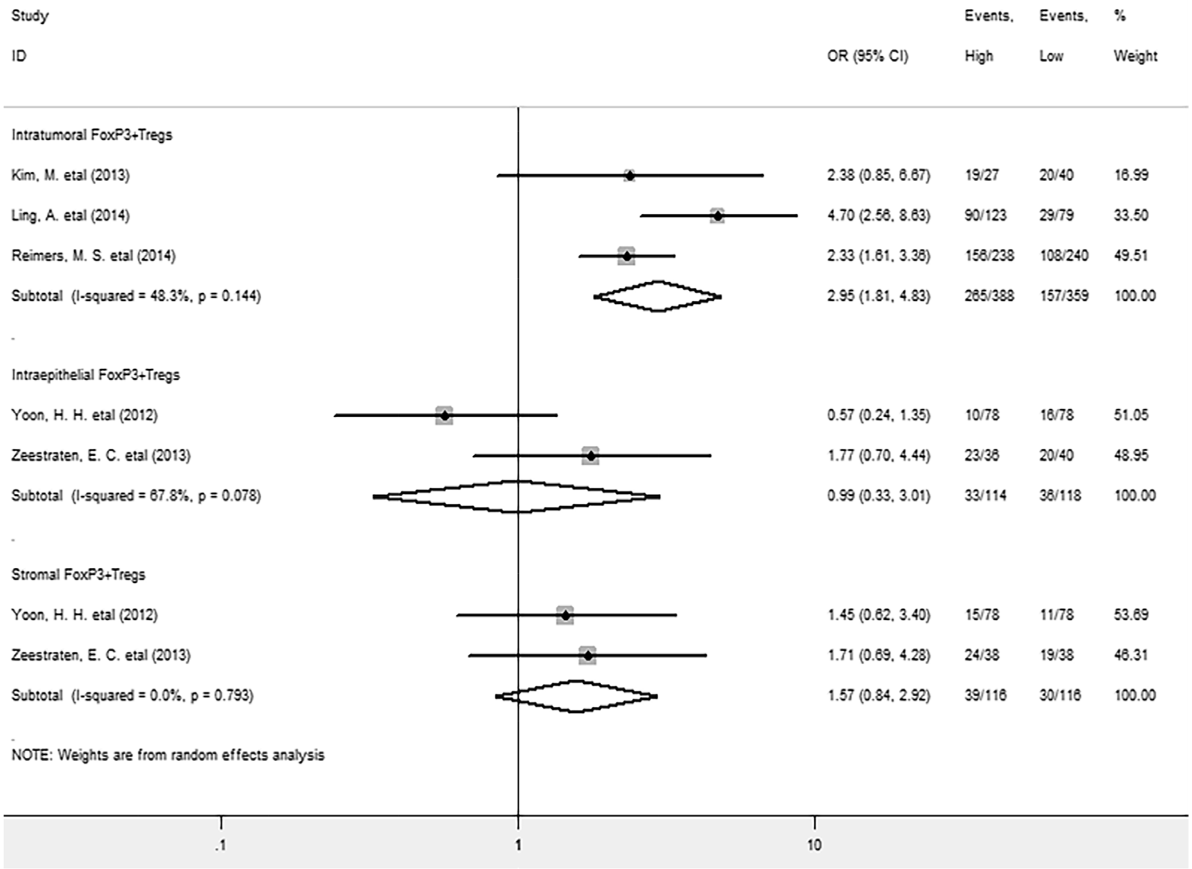

Supplementary Figure 3: Forest plots indicating ORs of the association between FoxP3^{+} Tregs from both stroma and intraepithelium and TNM stage.

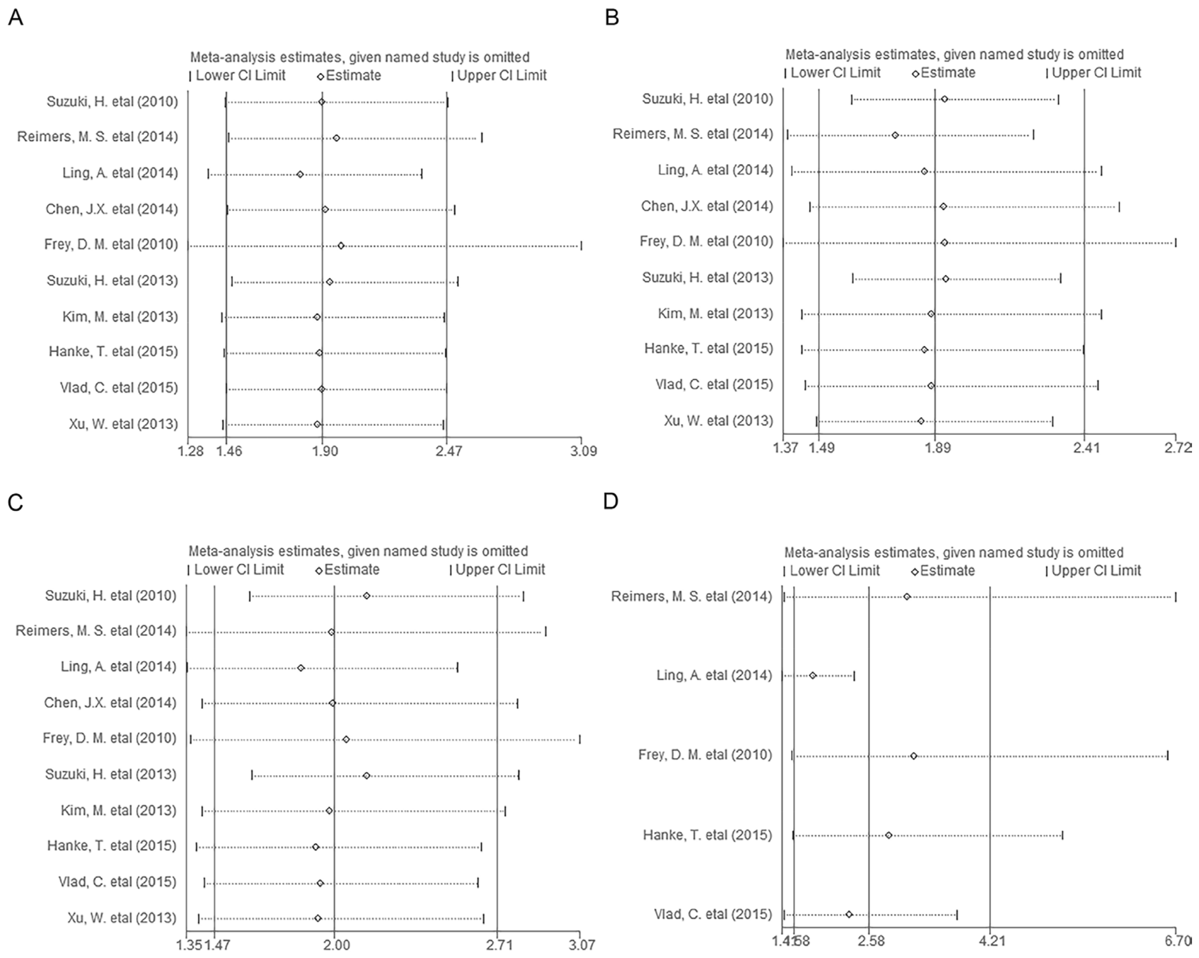

Supplementary Figure 4: Plots describing the influence of individual studies on the overall ORs for OS at 1, 3, 5 and 10-year **(A, B, C,** and **D)** respectively.

Supplementary Table 1: Characteristics of the included studies for OR analysis of clinicopathological features

Location of FoxP3$^+$ Tregs	Study	Year	No. of Patients	Cut-off for high density	FoxP3$^+$ Tregs: high / low	Tumor stage	I+II / III+IV	Quality Score (NOS)
Both intraepithelial and stromal compartments	Kim, M. etal [18]	2013	65	mean	27/38	I-IV	H: (19/8); L: (20/20)	7
	Ling, A. etal [12]	2014	204	other	125/79	I-IV	H: (90/33); L: (29/50)	8
	Reimers, M. S. etal [14]	2014	478	median	238/240	I-IV	H: (156/82); L: (108/132)	6
Intraepithelial compartment	Yoon, H. H. etal [27]	2012	78	other	78/78	II, III	H: (10/68); L: (16/62)	6
	Zeestraten, E. C. etal [24]	2013	76	median	36/40	I-III	H: (23/13); L: (20/20)	7
Stromal compartment	Yoon, H. H. etal [27]	2012	78	other	78/78	II, III	H: (15/63); L: (11/67)	6
	Zeestraten, E. C. etal [24]	2013	76	median	36/40	I-III	H: (24/14); L: (19/19)	7

T: primary tumor; N: lymph node; M: metastasis; H: high; L: low.
